# Relationships between predicted moonlighting proteins, human diseases, and comorbidities from a network perspective

**DOI:** 10.3389/fphys.2015.00171

**Published:** 2015-06-23

**Authors:** Andreas Zanzoni, Charles E. Chapple, Christine Brun

**Affiliations:** ^1^INSERM, UMR_S1090 TAGCMarseille, France; ^2^Aix-Marseille Université, UMR_S1090, TAGCMarseille, France; ^3^Centre National de la Recherche ScientifiqueMarseille, France

**Keywords:** moonlighting proteins, human disease, protein-protein interactions, multifunctional proteins, disease comorbidity

## Abstract

Moonlighting proteins are a subset of multifunctional proteins characterized by their multiple, independent, and unrelated biological functions. We recently set up a large-scale identification of moonlighting proteins using a protein-protein interaction (PPI) network approach. We established that 3% of the current human interactome is composed of predicted moonlighting proteins. We found that disease-related genes are over-represented among those candidates. Here, by comparing moonlighting candidates to non-candidates as groups, we further show that *(i)* they are significantly involved in more than one disease, *(ii)* they contribute to complex rather than monogenic diseases, *(iii)* the diseases in which they are involved are phenotypically different according to their annotations, finally, *(iv)* they are enriched for diseases pairs showing statistically significant comorbidity patterns based on Medicare records. Altogether, our results suggest that some observed comorbidities between phenotypically different diseases could be due to a shared protein involved in unrelated biological processes.

## Introduction

Moonlighting proteins are a special sub-class of multifunctional proteins performing multiple autonomous, often unrelated functions (Jeffery, 1999; Huberts et al., 2010; Copley, 2012). For instance, the human aconitase, encoded by the *ACO1* gene, is an enzyme of the tricarboxylic acid cycle (TCA cycle) which turns into a translation regulator upon a conformational change, when the iron concentration is low (Volz, 2008). Such switch between functions can molecularly depend on changes in substrates, post-translational modifications, the action of effector molecules, and other physiochemical factors. They may, in some cases, modify the oligomerization or conformational status of the protein. Moreover, additional functions may arise from a shift in the sub-cellular localization, the physicochemical environment or the pathological status of the cell, or a combination thereof (Jeffery, 2014).

In disease states, the switch of function of a moonlighting protein may occur in different ways. First, moonlighting proteins can be “neomorphic” (Jeffery, 2011), meaning that the protein does not play the same role in healthy and disease conditions. Therefore, the secondary function of the protein is solely revealed during the progression of a pathological state. This is illustrated by HMMR, a nuclear microtubule-associated protein in normal cells, which is exported to the extracellular matrix in certain tumors, where it binds the CD44 antigen, activates the ERK pathway, and ultimately promotes metastasis (reviewed in Maxwell et al., 2008; Jiang et al., 2010). Second, the different functions performed by a moonlighting protein in a healthy organism can be impaired by the effect of mutations in the disease state, often leading to complex phenotypes. For example, the phosphoglucose isomerase (PGI) is a moonlighting protein in healthy conditions. It converts Glucose 6-phosphate to Fructose 6-phosphate and also functions as a neurotrophic factor (Haga et al., 2000). Mutation leading to the inactivation of both molecular functions provokes a hemolytic anemia with neurological defects whereas a mutation impairing only the enzymatic function causes solely hemolytic anemia (Kugler et al., 1998). This naturally raises the question of a possible relationship between moonlighting proteins and the co-occurrence of different diseases within a single individual, i.e., disease comorbidity. Interestingly, recent works suggest that disease comorbidity generally occurs due to the dys-regulation of a common underlying cellular process through shared genes or protein interactions (Goh et al., 2007; Park et al., 2009). Hence, in the case of diseases that share a moonlighting protein, we hypothesize their co-occurrence may be caused by the dysregulation of distinct cellular processes.

As a first step toward the large-scale identification of moonlighting proteins, we have recently developed a pipeline that can detect what we have termed “extreme multifunctional proteins” (EMFs) from a protein-protein interaction (PPI) network (Chapple et al., 2015). These are proteins whose multiple functions are very different to one another. Although, EMFs will not all necessarily adhere to the strict definition of a moonlighting protein in term of protein organization, that is performing several unrelated functions without partitioning them between different several protein domains, (for further discussions see Jeffery, 1999; Huberts et al., 2010; Copley, 2012; Chapple et al., 2015), they will be however enriched in moonlighting candidates. Our analysis showed that 30% of the human interactome consists of multifunctional proteins, 10% of which are candidate EMFs. We demonstrated that they form a distinct sub-group of proteins characterized by specific features, constituting a signature of extreme multifunctionality (Chapple et al., 2015).

Here, we further investigate their involvement in human disorders and their possible role in disease comorbidities. Indeed, we found that EMF candidates appear to be involved in more than one disease, to contribute to complex rather than monogenic diseases, and to be involved in diseases leading to different phenotypes according to their annotations, when they are compared to other non-candidates proteins. Finally, they are implicated in diseases showing statistically significant comorbidity patterns based on MediCare records (Park et al., 2009). Overall, these results suggest that a protein involved in unrelated cellular processes can be implicated in phenotypically different diseases, therefore providing a possible molecular explanation for some known comorbidities between diseases observed at the level of individuals and populations.

## Materials and methods

### A dataset of EMF proteins

A dataset of 412 EMF candidates (Chapple et al., 2015) have been identified from a human protein-protein interaction network containing 74388 interactions between 12865 proteins, constructed by gathering interaction data from several databases through the PSICQUIC query interface (Aranda et al., 2011) (February 2013). Overlapping clusters have been extracted from the network using OCG (Becker et al., 2012), and annotated with Gene Ontology terms (Biological Process) using a majority rule. The EMF candidates are selected as proteins belonging to clusters annotated to dissimilar GO terms. GO term dissimilarity was assessed using two metrics of GO term co-occurrence, one based on the frequency of two terms annotating the same protein and the other on the frequency of interactions between proteins annotated to one term and those annotated to the other. These metrics allow the identification of functions that are very rarely performed by (i) a single protein and (ii) by interacting proteins, two proxies we consider indicators of *unrelated functions* (Chapple et al., 2015).

### Disease annotations

OMIM (Amberger et al., 2009) disease-protein associations and non-synonymous mutations were obtained from the UniprotKB database (Magrane and Consortium, 2011). OMIM disease phenotypes were mapped to Human Phenotype (Köhler et al., 2014) (HPO, “organ abnormality” branch) and Disease Ontology (Osborne et al., 2009) (DO) terms using their respective OBO ontology files. The broad HPO phenotype categories were defined using SimCT, a tool for ontology clustering (Herrmann et al., 2009).

### Phenotypic similarity measures

For each disease pair *a*-*b* associated with every protein of interest, we computed the phenotypic similarity of their HPO annotations using the Sørensen–Dice coefficient defined as:

$$PhenoSim_{S-D}=\frac{\text{2}(A\cap B)}{A\cup B}$$

and the Jaccard index defined as:

$$PhenoSim_{j}=\frac{A\cap B}{A\cup B}$$

where *A* is the list of HPO terms associated to disease *a*, and *B* the list of HPO terms associated to disease *b*.

### Disease semantic similarity

We used the DOSE package for R (Yu et al., 2015) for computing semantic similarities among Disease Ontology (DO) terms associated with every disease pair by applying both Lin's information content-based method and Wang's graph-based method (for a review, see Gan et al., 2013).

### Comorbidity data

The list of disease pairs with statistically significant comorbidity patterns and linked by gene sharing was taken from Park et al. (2009).

### Pfam domain detection

We used the Pfamscan tool to identify Pfam domains (version 26.0) (Punta et al., 2012). We kept only Pfam-A matches with an *E* < 10^−5^.

## Results

### A dataset of EMF candidates in the human interactome

EMFs, including moonlighting proteins, are expected to perform their independent functions through different interaction partners. In a recent work (Chapple et al., 2015), we have identified EMF protein candidates from the human interactome using OCG (Becker et al., 2012), an algorithm that identifies overlapping protein clusters in an interaction network. The cellular functions in which protein clusters are involved have been defined with regards to the Gene Ontology Biological Process annotations of their constituent proteins. Then, EMF candidates are proteins found at the intersection of clusters involved in very dissimilar cellular functions (see Methods section for details). Using this approach, we have identified two distinct categories of multifunctional proteins in the human interactome: 412 EMF candidates (MF-CAN) and 3434 non-EMF multifunctional proteins (MCNC for multi-clustered non-candidate). A third category consists of the remaining 9019 proteins in the interactome, which belong to only one cluster (MONO for mono-clustered). The proteins belonging to the three categories are listed in Supplementary Data Sheet 1. We have shown that the EMF candidates form a distinct sub-group of proteins displaying specific features distinguishing them from non-candidates proteins and constituting a signature of extreme multifunctionality. Among the striking features, EMF candidates are more connected in the network, enriched in short linear motifs (SLiMs) and in disease-related proteins compared to non-candidates, and are less intrinsically disordered than network hubs (Chapple et al., 2015).

### EMF candidates are enriched in proteins involved in more than one disease

**Figure 1:**
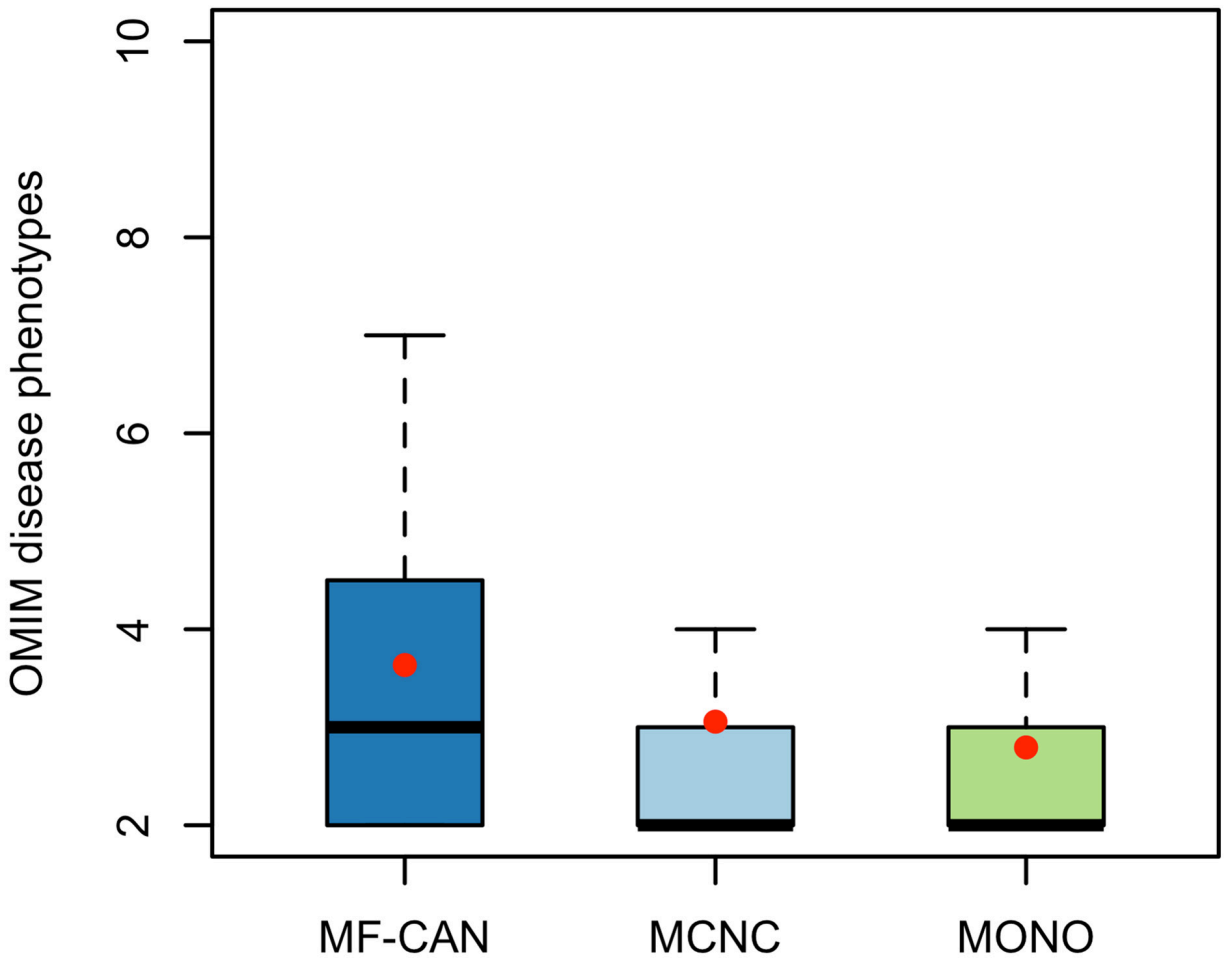
**Number of diseases associated with each protein category**. MF-CAN proteins involved in at least two diseases are associated with more diseases compared to MCNC (*P* = 2.6 × 10^−3^, Mann-Whitney U test, one-sided) and MONO proteins (*P* = 1.3 × 10^−4^, Mann-Whitney U test, one-sided). Mean values are depicted by red dots.

Using OMIM disease annotations, we found that the multifunctional proteins of the human interactome (i.e., proteins belonging to more than one network cluster, corresponding to MF-CAN and MCNC taken together) are enriched in proteins involved in diseases (1.33-fold, *P* < 2.2 × 10^−16^, Fisher's exact test, two-sided). We still observed this enrichment when considering MF-CAN and MCNC separately (Table 1). In addition, MF-CAN and MCNC are both significantly enriched in proteins involved in more than two diseases, with MF-CAN showing a higher proportion of such proteins compared to MCNC (2.04-fold and 1.25-fold, respectively). On the other hand, the MONO category is significantly depleted in disease proteins (Table 1). Given these results, we decided to focus our attention on those proteins of the three categories that are involved in more than two diseases. Interestingly, MF-CAN proteins are associated with a higher number of diseases compared to the other categories (3.6 compared to 3 and 2.9, on average, for MCNC and MONO respectively, *P* = 2.6 × 10^−3^ and *P* = 1.3 × 10^−4^, Mann-Whitney U test, one-sided) (Figure 1). This suggests that MF-CAN proteins are particularly associated to multiple diseases.

**Table 1:** **Proteins in the human interactome involved in OMIM diseases**.

**Set**	**Total number**	**At least one disease**	**At least two diseases**
MULTI	3846	830 (1.33, *P* < 2.2 × 10^−16^)	298 (1.34, *P* = 3.8 × 10^−7^)
MF-CAN	412	113 (1.84, *P* = 8.2 × 10^−8^)	52 (2.04, *P* = 1.8 × 10^−4^)
MCNC	3434	717 (1.28, *P* = 9.1 × 10^−12^)	246 (1.25, *P* = 6.8 × 10^−4^)
MONO	9019	1365 (0.87, *P* < 2.2 × 10^−16^)	349 (0.82, *P* = 3.8 × 10^−7^)

*Enrichment ratios (ER) and P-values for over- (ER > 1) and under-representation (ER < 1) are reported between parentheses (Fisher's Exact test, two-sided, alpha = 0.05). See main text for details*.

### EMF protein candidates contribute to complex diseases

**Figure 2:**
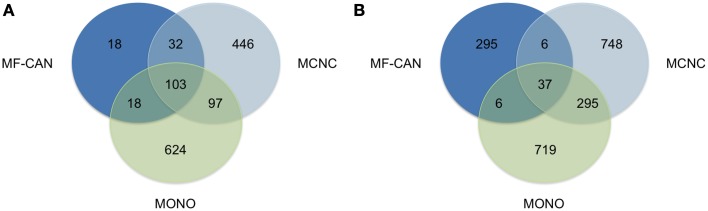
**Shared disease phenotypes and pairs among MF-CAN, MCNC, and MONO proteins**. **(A)** Most of the MF-CAN proteins are annotated with OMIM disease annotations also present in the other categories, whereas **(B)** they are associated with specific combination of diseases.

We next sought to verify whether MF-CAN, MCNC, and MONO proteins are associated with the same diseases. Figure 2A shows that most of the diseases (89%) in which the MF-CAN are involved, are also associated with at least one protein belonging to one of the other categories (compared to only 34% and 26% for MCNC and MONO, respectively). On the other hand, 86% of the disease *pairs* associated with MF-CAN are specific to this category (compared to ~68% for the other two categories) (Figure 2B). This, therefore, suggests that our EMF candidates are mainly involved in specific combinations of diseases. Moreover, the fact that diseases associated to MF-CAN are associated with several other genes/proteins as well (Figure 2A) suggests that EMF candidates are implicated in combinations of complex diseases rather than monogenic ones.

### EMF candidates are involved in phenotypically dissimilar diseases

Different OMIM diseases caused by the dysregulation of a same gene can affect the same body organs or have similar phenotypic manifestations (Goh et al., 2007), or not. We therefore tested this on the disease pairs associated with MF-CAN, MCNC, and MONO proteins.

**Figure 3:**
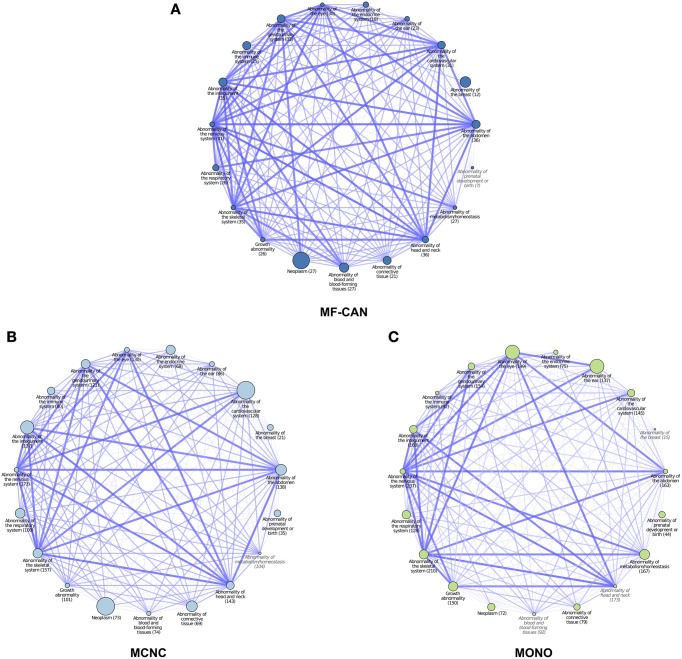
**Network representation of Human Phenotype Ontology (HPO) classes associated with MF-CAN (A), MCNC (B), and MONO (C) proteins**. Each node corresponds to a HPO broad phenotype class (organ abnormalities branch), the size of the nodes is proportional to the over-representation of proteins of the three categories among the proteins annotated to the node's phenotype. Nodes whose name is in gray and italic had no significant overrepresentation (*p* ≥ 0.05). The numbers in parentheses are the number of proteins of each category found annotated to each HPO class. Edges between nodes indicate proteins that are annotated to both HPO classes. The width and different color shade of the edge is proportional to the number of proteins shared by both HPO classes.

**Figure 4:**
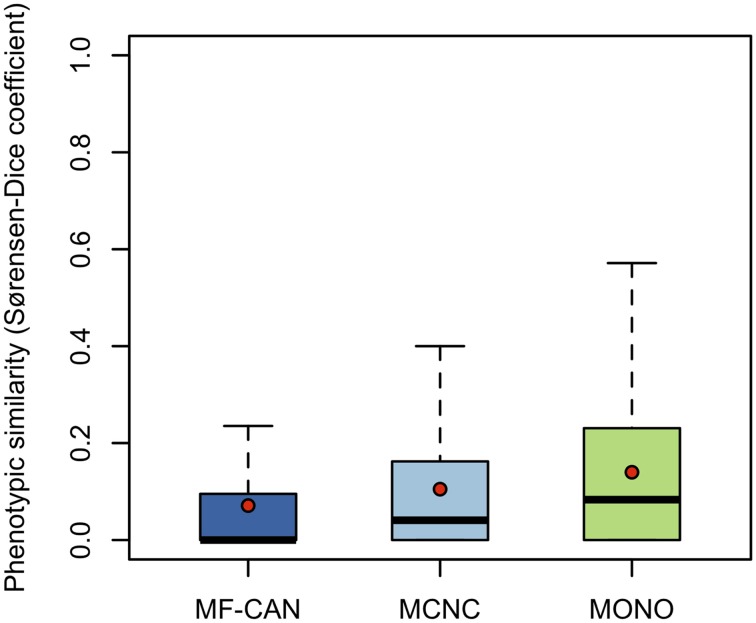
**Disease pairs phenotypic similarity based on Human Phenotype Ontology annotations**. Distributions of phenotypic similarity, measured using the Sorensen-Dice coefficient, for MF-CAN, MCNC, and MONO proteins. Mean values are depicted by red dots.

To do so, we mapped OMIM diseases to the Human Phenotype Ontology (HPO) (Köhler et al., 2014). For each disease, HPO provides a list of annotation terms describing the phenotypes associated to the disease. We clustered them in 19 broad classes of organ abnormalities. We found that proteins belonging to MF-CAN, MCNC, and MONO are over-represented in almost all of the HPO broad classes (Figure 3). Particularly, MF-CAN proteins are over-represented in all HPO classes except “abnormality of prenatal development or birth” (Figure 3A), MCNC proteins are under-represented in the “abnormality of metabolism/homeostasis” class (Figure 3B) whereas MONO proteins are under-represented in three HPO classes: “abnormality of the breast,” “abnormality of the blood and blood-forming tissue,” and “abnormality of head and neck” (Figure 3C). More interestingly, we observed a significantly higher fraction of shared MF-CAN proteins, 32% on average, between HPO classes compared to MCNC (21%, *P* = 2.3 × 10^−13^, Mann-Whitney U test, one-sided) and MONO (19%, *P* < 2.2 × 10^−16^). This result shows that MF-CAN proteins are involved in disease affecting distinct body parts. Therefore, this led us to evaluate the phenotypic similarity between disease pairs by computing the Sorensen-Dice distance based on their HPO annotation terms (see Methods for details). For each pair of diseases in which a same gene is involved, we have compared their phenotypic descriptions to assess their similarity aiming at grasping possible differences. We indeed found that those pairs associated with MF-CAN are significantly different (Figure 4, Supplementary Figure 1) compared to the other categories (*P* = 4.7 × 10^−5^ and *P* = 1.3 × 10^−11^ for MCNC and MONO, respectively, Mann-Whitney U test, one-sided). We obtained similar results using semantic similarity measures (Supplementary Figure 2) based on DO annotations (Schriml et al., 2012). Indeed, MONO disease pairs are significantly more similar than MF-CAN and MCNC pairs (*P* = 2.7 × 10^−3^ and 1.5 × 10^−5^, respectively, Mann-Whitney U test, one-sided). These results could be due to the fact that multifunctional candidates (MF-CAN and MCNC) are acting in several biological processes, and are therefore involved in different diseases and phenotypes.

Diseases leading to different phenotypes could be due to mutations located in different protein regions. Given the strict definition applied to moonlighting protein which should perform their multiple functions without partitioning these into multiple domains (Jeffery, 1999), we sought to verify whether mutations causing different diseases are located within the same functional domains. For this, we compared the phenotypic similarities of disease pairs whose mutations are either found in the same Pfam domains or outside. Interestingly, MF-CAN proteins show a lower phenotypic similarity than MCNC and MONO in both situations (Supplementary Figure 3). Therefore, diseases in which MF-CAN are involved, display different phenotypes irrespective to the location of the corresponding mutations.

Overall, these results show that MF-CAN are involved in diseases leading to different phenotypes and affecting different organs.

### EMF candidates are involved in comorbid diseases

In order to estimate whether moonlighting candidates could explain some comorbidity patterns, we used the list of comorbid diseases established by Park et al. (2009), based on Medicare records. We found (Table 2, Supplementary Data Sheet 2) that EMF candidates are enriched in proteins involved in at least one disease pair showing statistically significant comorbidity patterns (*P* = 3.5 × 10^−6^, Fisher's exact test, two-sided), whereas MONO are significantly depleted in such pairs (*P* = 1.1 × 10^−5^).

**Table 2:** **Number of proteins associated with at least one disease pair showing comorbidity patterns**.

**Set**	**Total number**	**Disease comorbidity**
MF-CAN	52	27 (1.95, *P* = 3.5 × 10^−6^)
MCNC	246	67 (1.25, *P* = 0.0858)
MONO	349	58 (0.52, *P* = 1.1 × 10^−5^)

*Enrichment ratios (ER) and P-values for over- (ER > 1) and under-representation (ER < 1) are reported between parentheses (Fisher's Exact test, two-sided, alpha = 0.05)*.

Our results therefore indicate that EMF proteins involved in several diseases can contribute to comorbidity, probably through the unrelated biological processes in which they participate.

### An example of EMF candidate involved in a pair of comorbid diseases

Androgen receptor (AR) is one of the MF-CAN proteins associated to a disease pair showing a comorbidity pattern (Supplementary Data Sheet 2): prostate cancer (PC) and spinal and bulbar muscular atrophy (SBMA, ICD-9: 335.1), an illness leading to progressive muscle weakness and atrophy, known also as Kennedy's disease. SBMA is a late-onset neurodegenerative disease, caused by an extreme expansion of trinucleotide CAG repeats in the AR gene first exon, which encodes for a stretch of glutamines (polyQ) in the N-terminal transactivation domain of the AR protein (Kumar et al., 2011). Interestingly, *in vivo* studies on mice harboring humanized AR alleles showed that long polyQ may be involved in late-onset PC and influence hormone sensitivity (Albertelli et al., 2008; Simanainen et al., 2011). Recently, a case of SBMA and PC comorbidity has been reported in the literature (Kosaka et al., 2012). Altogether, these observations are consistent with the comorbidity reported in Medicare, since it collects hospital visit reports of elderly patients (age equal or above 65). Interestingly, polyQ stretches induce the formation of AR aggregates within neuronal cells, thus impairing AR interaction ability. We can therefore speculate that AR aggregates in prostate cell can have a similar effect, probably by perturbing a different set of AR interactions.

## Discussion

Network approaches recently suggested that the co-occurrence of different diseases within the same individual, i.e., comorbidity, correlates with the sharing or the interactions of their underlying molecular components in cellular networks (Park et al., 2009; Zhou et al., 2014). Nevertheless, these molecular components can also be involved in distinct cellular processes. Since moonlighting, and by extension EMF, proteins are implicated in unrelated cellular processes (Jeffery, 1999; Copley, 2012), often taking places in different subcellular localizations, we postulate that disease comorbidities could be due not only to the dysregulation of shared underlying sub-networks as previously proposed, but also to the dysregulation of different ones in which a shared protein, able to moonlight, is taking part. To test this hypothesis, we investigated the potential involvement of a particular group of proteins, the EMF candidates, in diseases. We show that EMF candidates are involved in multiple diseases that are phenotypically dissimilar and affect distinct organs.

Moreover, we found that a significant fraction of these EMF candidates represents a possible molecular link between comorbid diseases. Nevertheless, although a gene/protein may be involved in different diseases, these diseases are not necessarily co-occurring within a single individual. Our finding that comorbid diseases identified from Medicare records are enriched among EMF candidates therefore reinforces *(i)* the fact that diseases in which an EMF is involved can occur in a same individual and *(ii)* our hypothesis that comorbidity can be due their involvement.

Diseases associated to EMF proteins display different phenotypes according to HPO, independently of the location of the mutations in the protein sequence, within or outside known protein domains. This raises several possibilities. First, these mutations may occur in existing domains not yet discovered. Indeed, current tools may not have detected some domains, since they are below the detection thresholds, or differing from the model used by the prediction tools. Second, this lack of difference may also be explained by the fact that disease mutations occur in SLiMs, short stretches of amino acids sequences that represent potential regulatory sites and are located mostly in intrinsically disordered regions (van Roey et al., 2013). As *(i)* we previously showed that EMFs are enriched in SLiMs compared to MCNC (Chapple et al., 2015) and *(ii)* a recent report showed that disease mutations occur more likely within SLiMs than neutral missense mutations in intrinsically disordered regions (Uyar et al., 2014), the second hypothesis mentioned above is thus supported.

In an early study on co-occurring metabolic diseases, Barabasi and colleagues (Lee et al., 2008) proposed that the lack of observed comorbidities between 69% of the metabolic disease pairs they investigated, could be due to moonlighting enzymes involved in non-metabolic functions. Indeed, the corresponding subnetworks were missing from the metabolic network they were investigating. Interestingly, for 95% of the EMF, one of the functions is related to various metabolic processes whereas the other is non-metabolic, such as signaling, transport, or localization (Table 1 in Chapple et al., 2015). Therefore, given the link between EMFs and moonlighting explained in the Introduction, our results support the hypothesis that moonlighting proteins could indeed provide the missing connections between metabolic diseases and the rest of the disease network (Goh et al., 2007).

It should be noted that our observations cross and link several integrated functional levels (Brun et al., 2004; Barabási, 2007). First, the disease pairs in which EMF candidates play a role are the most dissimilar in terms of phenotype. Since candidates have been selected as involved in unrelated cellular functions, this observation directly links cellular processes to disease phenotypes, based on the analysis of independent annotations (Gene Ontology and Human Phenotype Ontology). It thus appears that for these proteins, noticeable differences are observed from the molecular level (mutations) to the cellular level (unrelated processes) up to the tissular/organismal level (phenotypes), thus emphasizing the complexity of the link between genotype and phenotype.

Finally, grounded on our computational identification of moonlighting candidates, this work represents, to our knowledge, the first effort toward the elucidation of the convoluted relationships between moonlighting protein functions, disease phenotypes and comorbidities. Overall, although much remains to be learned, our analysis underlined the necessity of identifying moonlighting proteins and to better understand their role in human diseases, particularly in the context of drug development to avoid side effects. Indeed, knowing that the different diseases in which a protein is involved, are due to the dysregulation of different processes may help choosing specific interactions to inhibit in order to act on one disease and not the other. In addition, a drug inhibiting an unsuspected moonlighting protein may cause side effects if it also impairs the secondary function of the protein. Therefore, the identification of candidate moonlighting proteins can be useful to gain a deeper understanding of disease molecular details and to design more effective therapeutic strategies.

### Conflict of interest statement

The authors declare that the research was conducted in the absence of any commercial or financial relationships that could be construed as a potential conflict of interest.
