# Supplementary material for: Relationships between predicted moonlighting proteins, human diseases, and comorbidities from a network perspective
Source: Front Physiol. 2015 Jun 23;6:171. doi: 10.3389/fphys.2015.00171 (PMC4477069; doi:10.3389/fphys.2015.00171)
Supplement: Supplementary file 3 [file Image1.PDF]

Supplementary Material

Relationship between predicted moonlighting proteins, human diseases and comorbidities from a network perspective.

Andreas Zanzoni [1,2], Charles E. Chapple[1,2], Christine Brun[1,2,3]*

[1]Inserm, UMR_S1090 TAGC, Marseille, F-13288, France
[2]Aix-Marseille Université, UMR_S1090 TAGC, Marseille, F-13288, France
[3]CNRS, Marseille, F-13402, France.

* **Correspondence:** Christine Brun, Aix-Marseille Université, TAGC, Marseille, France; INSERM UMR_S1090, Marseille, France. brun@tagc.univ-mrs.fr

1. Supplementary Figures

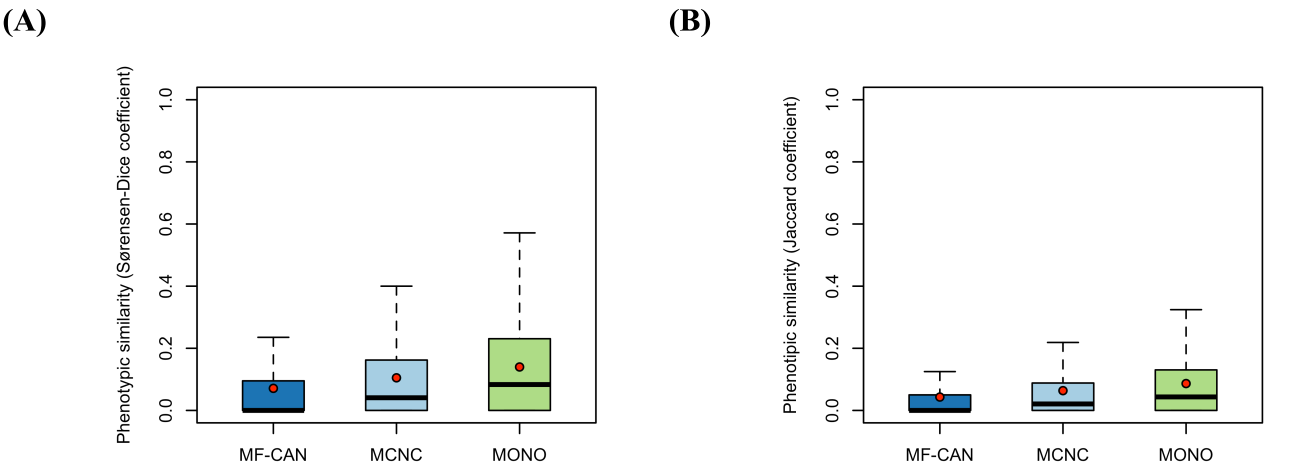

Supplementary Figure 1. Disease pairs phenotypic similarity based on Human Phenotype Ontology annotations. For MF-CAN, MCNC and MONO proteins: (A) distributions of phenotypic similarity measured using the Sorensen-Dice coefficient (same as Figure 4A in the main text); (B) distributions of phenotypic similarity measured using the Jaccard index. Mean values are depicted by red dots.

(A) **(B)**

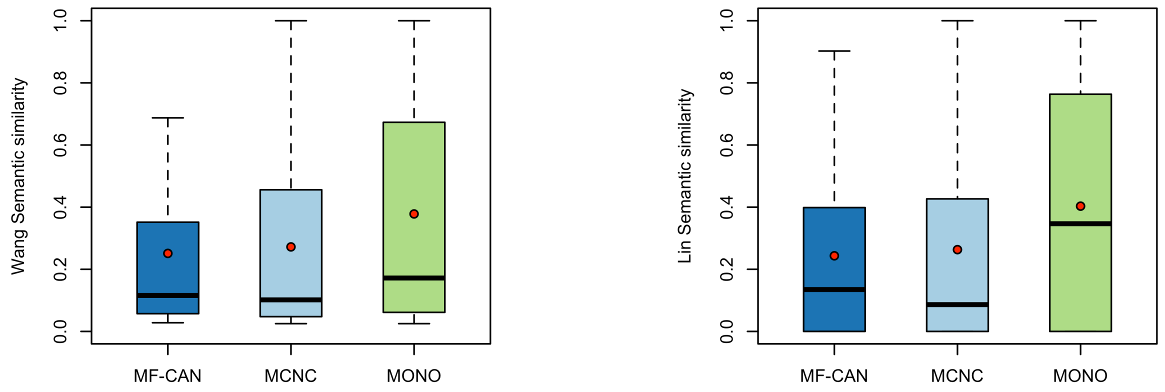

Supplementary Figure 2. Disease pairs phenotypic similarity based on Disease Ontology annotations. (A) MF-CAN Wang semantic similarity distribution does not significantly differ from MCNC (P-value=0.7598, Mann-Whitney U test, one-sided), but it is significantly different when compared to MONO (P-value= $2.7x10^{-3}$, Mann-Whitney U test, one-sided) protein sets showing lower means. The similarity distribution of MCNC is also significantly different compared to MONO (P-value= $1.5x10^{-5}$, Mann-Whitney U test, one-sided). (B) distributions of phenotypic similarity measured using the Lin semantic similarity. No significant difference is found between MF-CAN and MCNC disease pairs (P-value=0.6496, Mann-Whitney U test, one-sided). Both MF-CAN and MCNC similarity distribution are significantly different compared to MONO (P-value=$4.0x10^{-3}$ and P-value=$2.4x10^{-4}$ respectively, Mann-Whitney U test, one-sided). Mean values are depicted by red dots.

(A) (B)

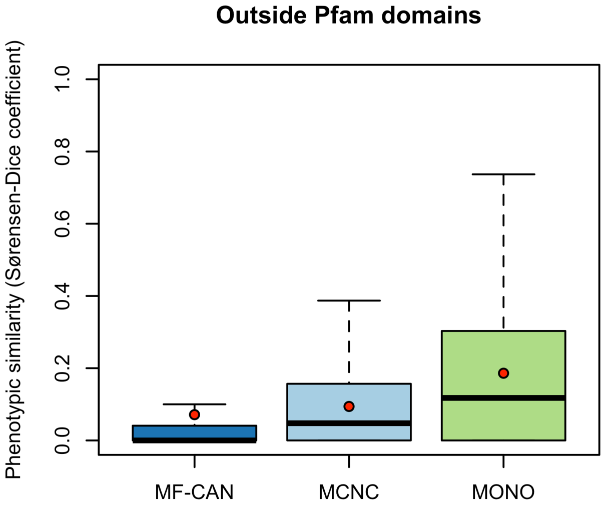

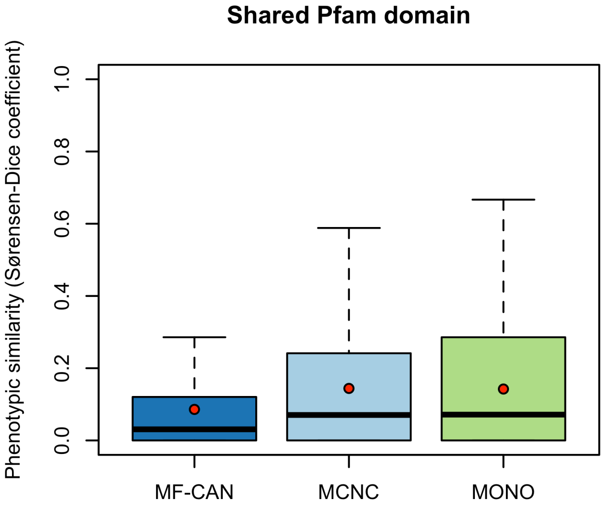

Supplementary Figure 3. Disease pairs phenotypic similarity based on Human Phenotype Ontology annotations. The phenotypic similarity distributions of Figure 4 in the main text were split considering whether the two diseases have mutations in the same Pfam domains or not (A) MF-CAN disease pairs with mutations outside Pfam domains are significantly less similar compared to MCNC and MONO (P-value=6.0×10^{-3} and P-value=2.7×10^{-6} respectively, Mann-Whitney U test, one-sided). MCNC are also less similar compared to MONO (P-value=8.0×10^{-7}, Mann-Whitney U test, one-sided). (B) Similarly, MF-CAN disease pairs whose mutations lie within the same Pfam domain are significantly less similar when compared to MCNC and MONO (P-value=9.8×10^{-3} and P-value=2.2×10^{-2} respectively, Mann-Whitney U test, one-sided). In this case no significant difference is observed between MCNC and MONO (P-value=0.5657, Mann-Whitney U test, one-sided). Mean values are depicted by red dots.
